# Preeclampsia in 2018: Revisiting Concepts, Physiopathology, and Prediction

**DOI:** 10.1155/2018/6268276

**Published:** 2018-12-06

**Authors:** J. Mayrink, M. L. Costa, J. G. Cecatti

**Affiliations:** Obstetric Unit, Department of Obstetrics and Gynecology, School of Medical Sciences, University of Campinas, Campinas, Brazil

## Abstract

Preeclampsia currently remains one of the leading causes of death and severe maternal morbidity. Although its prevalence is still underestimated in some places due to underreporting, preeclampsia is a disease that health professionals need to know how to deal with and take action. For this reason, the studies about the theme remain along with the advances in their understanding that often implies improvement and change of concepts and conducts. The complexity of its etiology is a challenge and requires further studies for its full understanding. Apparently, poor adaptation of the maternal organism to the conceptus, marked by the nonoccurrence of changes in the uterine spiral arteries, determines a series of systemic repercussions that compound the various forms of preeclampsia presentation. In recent years, the use of acetylsalicylic acid to prevent cases of early onset of the disease has been consolidated and, alongside, studies have advanced the development of accessible and effective methods of identifying women at risk of preeclampsia. The aim of this review is to discuss updates on the occurrence, concept, pathophysiology, repercussion, prevention, and prediction of preeclampsia.

## 1. Introduction

According to some German authors, the first reports referring to eclampsia date from 2200 BC, observed in papyri of ancient Egypt [1]. The word eclampsia originates from the Greek* éklampsis* and means “bright light” [1]. For about 2000 years, eclampsia was understood as a disease characterized by convulsive seizures, typical of late gestation, that ended at the childbirth. Scientists from the late 19th century, true enthusiasts of caregiver empiricism, recognized the similarity between the swollen appearance of women who had seizures and the edema of Bright's disease, an abrupt glomerulonephritis onset characterized by proteinuria. Thereafter, urinary alterations in childbearing women with seizures were searched, which culminated in finding proteinuria in them. With the advent of noninvasive blood pressure measurement, it was observed that these women had increased blood pressure levels. It was not long before the understanding came that proteinuria and arterial hypertension preceded the onset of the seizures. Thus, it was defined the “preeclampsia” hypertensive condition, already understood at the time in its progressive character of severity and which could lead, and led in a grim way, to series of consequences for the maternal and fetal lives [1–3].

Even today, hypertensive conditions portray important complications during the gestation, with an incidence that varies according to the particularities of the population studied, and can exceed the mark of 10% in some regions [4]. Preeclampsia and eclampsia rank second or third in the world ranking of maternal morbidity and mortality causes [5]. In an analysis implemented by the World Health Organization, which evaluated the causes of maternal death occurred between 2003 and 2009, the hypertensive causes appear in the second place, occurring in 14% of the cases, preceded only by hemorrhagic causes, responsible for 27.1% of the maternal deaths [6].

Recently,* Abalos et al*., in a systematic review involving 40 countries with 39 million women, showed an estimated rate of preeclampsia and eclampsia of 4.6% and 1.4%, respectively; in Brazil, these numbers were 1.5% and 0.6%. The review mentioned countries where these numbers are not even known due to lack of official records, which makes it very difficult to recommend strategies for interventions that could contribute to better maternal and perinatal outcomes [7]. Despite the numbers presented, Brazilian data are still underestimated because there are unquestionable regional differences in a continental sized country, as* Giordano et al.* have demonstrated in finding eclampsia prevalence of 0.2% in South and Southeast regions while in the North, Northeast, and Center-West regions, this proportion was of 8.1% [8].

Still regarding Brazil, according to data published in an analysis of 2016, in which more than 80,000 women (from the five regions of the country) were monitored for severe maternal morbidity, it was observed that the main cause of hospital admission was hypertension, making up 73% of that classified as maternal life-threatening condition, given the fact that the main cause of maternal death in the country is eclampsia. The mortality index (calculated by the ratio of maternal death cases to maternal near miss + maternal death) found at that time was 15.4%, which is considered an acceptable value (below 20%), but still very high, far from the ones observed in high-income countries (<2%) [9]. In the analysis of eclampsia cases, considering the same study of severe maternal morbidity, the prevalence of near-miss events (near miss, the most extreme form of severe maternal morbidity), including eclampsia, was of 4.2 cases per 1000 live births or 8.3 cases per maternal death [10]. It is important to highlight the use of the near miss indicator or severe acute maternal morbidity, which includes the cases of women who almost died from some Potentially Life-Threatening Condition (PLTC) during the pregnancy-puerperal period but, nevertheless, survived. This analysis is able to reflect more accurately on the quality of obstetric care if compared to the absolute number of maternal deaths, which by itself is already a rare event. For this reason, in the last decade, there was an increase in interest in this indicator, which culminated in the World Health Organization's initiative to standardize maternal near miss case identification criteria, intending to facilitate the monitoring and planning of improvements in obstetric care [11].

In the last 50 years, there has been a decreasing trend in the incidence of these aggravations in high-income countries, alongside an opposite movement in middle- and low-income countries, which is basically due to access to quality prenatal care as well as adequate management of cases of preeclampsia and eclampsia, with better maternal and perinatal outcomes [7, 15]. Broadening the analysis in a generalized way to other poor obstetric outcomes, in order to illustrate this discrepancy in numbers in middle- and low-income countries, the risk of death due to maternal causes can reach up to 33 times that observed in high-income countries [16]. As it has already been demonstrated, a number of more than 8 prenatal visits is a protective factor for the occurrence of preeclampsia [17]. An inquiry conducted in California in 2011, in order to investigate the increase in maternal deaths observed in that region in the early 2000s, revealed that 79% of preeclampsia related deaths were due to poor management [18, 19]. In other words, the improvement of maternal and fetal health outcomes is absolutely associated with the wide access to services and the quality care and management of the complications, translated into better perinatal outcomes.

## 2. Concepts and Classification

Hypertensive conditions during pregnancy can be classified as arterial hypertension prior to gestation or with manifestation before 20 weeks and arterial hypertension starting at or after 20 weeks. The first group includes

- essential chronic or secondary arterial hypertension;
- white coat hypertension;
- “Masked” hypertension.

The hypertension group, which appears at 20 weeks or more, includes

- transient gestational hypertension;
- gestational hypertension;
- preeclampsia, which can be isolated or superposed on chronic hypertension. In this group, arterial hypertension is defined as systolic blood pressure equal to or greater than 140 mmHg and/or diastolic blood pressure equal to or greater than 90 mmHg, which should be measured on two distant occasions at least 4-6 hours apart, in a calibrated and adequate blood pressure monitor for the biotype of the woman under evaluation and managed by a trained professional [20]. When it comes to preeclampsia, one of the following conditions must coexist:
  - Proteinuria (demonstrated by the ratio of proteinuria/creatininuria above 0.3 mg/mg, or by urine dipstick test equal to or above 1+, or by 24-hour proteinuria above 300mg / 24h);
  - Dysfunctions of maternal organs which can be renal insufficiency, characterized by creatinine above 1.02 mg/dL; hepatic impairment, characterized by an elevation of transaminases two times above normal levels, or pain in the right hypochondrium, or epigastralgia; neurological complications, characterized by scotomas or persistent cephalgia accompanied by hyperreflexia or confusional states or eclampsia or cerebrovascular accident or amaurosis; and haematological complications consisting of thrombocytopenia or hemolysis;
  - Uteroplacental dysfunctions: fetal growth restriction; changes in the Doppler velocimetry studies of the umbilical artery, especially if combined with alterations in uterine arteries [20, 21].

As it can be noticed, proteinuria is not a* sine qua non* condition to characterize preeclampsia, as previously ensued [22, 23]. According to the concept proposed by the International Society for Studies in Gestational Hypertension, published in 2014 and reinforced in 2018 [24], every hypertensive pregnant woman should be investigated for multiple organ involvement, even if presenting negative proteinuria, in order to discard the hypothesis of preeclampsia. This approach is innovative and tends more broadly to encompass cases that are somewhat neglected by the absence of proteinuria.

Preeclampsia may show signs of severity when systolic blood pressure levels are greater than 160mmHg and/or diastolic blood pressure levels are greater than 110mmHg, or when there is concomitance of eclampsia or HELLP syndrome. The last is defined as hemolysis, thrombocytopenia with a platelet count inferior to 150,000, and elevation of hepatic transaminases twice the upper limit of normality [21]. Massive proteinuria (above 5 grams in 24 hours) was no longer considered an isolated criterion of severity with the conceptual modifications proposed by the International Society for Studies on Gestational Hypertension, in 2014 [25] and should now be evaluated in consonance with the other clinical data and laboratory tests presented by the pregnant woman in question, mainly to decide the ideal moment of gestational interruption.

Regarding the moment of manifestation, preeclampsia is called early when it occurs before completed 34 weeks of gestation and late after this gestational age. It may also be preterm when the onset occurs between 34 weeks and 1 day and 37 weeks and term preeclampsia when it occurs from 37 weeks and 1 day [21, 26]. The need of classifying preeclampsia is enforced as placental histological analysis of women with the aggravation showed that, in those with early onset, the vascular lesions were predominant and lower placental volume was evident, whereas larger placental volumes were more common in cases of manifestation after 37 weeks, with signs of chronic inflammatory response [27]. In this sense, cases of early-onset preeclampsia would be more associated with placental insufficiency and therefore with fetal growth restriction. On the other hand, later-onset cases would present milder clinical conditions: in summary, different placental damages and distinct phenotypes [3, 28–30].

## 3. Pathophysiology

The pathophysiology of preeclampsia has not yet been fully elucidated. It is likely to be elapsed from a model composed of two interrelated stages: abnormal placentation and maternal inflammatory response [31, 32].

The process known as placentation is meticulously coordinated, and the equilibrium maintenance of the fetoplacental unit depends on its effective occurrence. In a normal pregnancy, there is a considerable increase in uterine blood flow in order to ensure adequate supplementation for the intervillous space and, by extension, adequate fetal development. In order to achieve this result, spiral arteries undergo a process of remodeling composed of 4 sequential steps promoted by a trophoblastic invasion of their walls. Initially, the decidua is invaded, followed by intra-arterial trophoblast migration, with a subsequent intramural invasion of the vessels, when there is loss of the middle (muscular) layer, replaced by fibrinoid material and connective tissue. The last step is that of vessel reendothelialization and other induced maternal adaptations [30, 33]. These vessels start to present a mean diameter, much higher than that observed in uteri of nonpregnant women with low resistance to blood flow, and can thus provide intervillous space with adequate blood supply to maintain pregnancy effectively [34, 35] (Table 1). On the other hand, the radial and arched arteries will have increased blood pressure in their walls, resulting from the higher blood flow, which will act as a stress producer and, ultimately, will generate nitric oxide secretion by the endothelium, determining an overall vasodilation of the uterine vessels [35]. The remodeling of the spiral arteries occurs more in the central part of the placental bed, reducing progressively towards the periphery [30].

This process of adaptation of the spiral arteries cannot occur ideally when approximately 90% of the vessels undergo the expected changes. In pathological cases, remodeling may be partial, completely absent, or even absent with obstructive vessel lesions. In cases of preeclampsia, the proportion of remodeled vessels is found considerably reduced, especially in the central region of the placental bed. When associated with fetal growth restriction, there are obstructive lesions. In these cases, the arteries undergo a process of atherosis with very similar results to the formation of atheromatous plaques, with their lumens invaded by lipid-rich macrophages, perivascular mononuclear inflammatory infiltrate, and fibrinoid necrosis of the vessel walls, with consequent uteroplacental ischemia. Then, a vicious circle of ischemia and reperfusion in the intervillous space is installed, with the metabolic stress of the endoplasmic reticulum of the trophoblast cells, which are structures responsible for cellular homeostasis and, ultimately, for the apoptosis of the same. This process releases nanomolecules into the maternal circulation, which is capable of triggering a broad intravascular inflammatory response, an essential step for the development of preeclampsia [33], as well as free radicals, in consequence of the extensive oxidative stress and the collapse of placental mechanisms and antioxidants enzymes [36]. It can be said that such a process is immunomediated, as it involves systemic inflammatory response and maternal genetic predisposition [34, 36, 37].

The aforementioned oxidative stress, as well as the cellular apoptosis, would be determinant for an imbalance between proangiogenic and antiangiogenic factors, with a predominance of the latter [37]. Increased concentrations of VEGFR-1 (capable of blocking the angiogenic action of VEGF) and the soluble form of this vascular endothelial growth factor, sFlt-1(fms-like tyrosine kinase 1), a potent antagonist of VEGF action, and decreased synthesis of placental growth factor (PlGF) are associated with the predominance of antiangiogenic elements characteristic of preeclampsia [37, 38].

Finally, the understanding of the pathophysiology of preeclampsia, even though it is partial, includes the activation and consequent platelet consumption at levels above those observed in normal pregnancies, vasospasm, and prostacyclin deficiency that have a vasodilatory action and inhibit platelet aggregation. Otherwise, the synthesis of thromboxane A2 is increased in placentas of women with preeclampsia, which determines the predominance of vasoconstriction and increased platelet aggregation [39], as well as thrombin, which has its maximum expression in disseminated intravascular coagulation, clinically translated to placental abruption [40]. Bigger synthesis of thrombin is part of a more vigorous inflammatory response characteristic of preeclampsia, as already exposed above, and determines the deposition of fibrin in multiple organs, which reinforces the systemic character of the pathological condition.

## 4. Medium- and Long-Term Repercussions and Prevention

The diagnosis of preeclampsia or eclampsia in a pregnant woman is followed by efforts involving the possible acute implications of these disorders (severe thrombocytopenia, disseminated intravascular coagulation, placental abruption, among others). However, it is also very important to look upon their long-term implications, which are innumerable and, sometimes, irreparable. After a pregnancy complicated by preeclampsia, about 20% of women will develop hypertension or microalbuminuria within seven years, and the same occurs with only 2% of women who have had pregnancies with no complications [5]. Similarly, the risk of acute myocardial infarction, stroke, and venous thromboembolism is substantially higher in women with a personal history of preeclampsia, as demonstrated in a meta-analysis published in 2007 [41].

In relation to newborns, the complexity lies in the decision of the moment when the risks in the intrauterine environment outweigh the risks of the extrauterine one. In this sense, prematurity and its innumerable consequences, such as acute respiratory syndrome, intraventricular hemorrhage, sepsis, bronchopulmonary dysplasia, and deficits in neuropsychomotor development, are some of the scenarios with which the infant born from a mother with preeclampsia (usually premature, before 34 weeks) will have to come across and, very frequently, against which they will have to fight [20]. Some studies have already shown a negative impact on the neurocognitive development of these infants evaluated in their first two years of life [42, 43].

Ultimately, mothers who have experienced near-death experience (near miss, the most extreme form of severe maternal morbidity) because of preeclampsia may also have psychological consequences with emotional impacts that involve anxiety, isolation, difficulties in breastfeeding, depressive disorders, and impairment of reproductive capacity, among others. The prolonged stays in intensive care units, because of either the woman herself or the newborn, and physical or mental limitations may interrupt the natural order of the symbiosis between mother and baby [44].

In this context of multiple and devastating consequences of preeclampsia, the need for its prevention emerges. It has already been demonstrated by a meta-analysis that early use (before the 16th week of pregnancy) of low-dose aspirin reduces the occurrence of preeclampsia, especially in its more severe forms (before 34 weeks) [45]. A recent clinical trial involving about 2,000 pregnant women compared aspirin and placebo use and observed a 62% reduction in the occurrence of early preeclampsia, in the group that daily took 150 mg aspirin [46]. Metformin has emerged as a target of studies in different groups of pregnant women, regarding its effects on preeclampsia risk [47]. Recently, a meta-analysis showed that, compared to insulin, metformin decreased the risk of pregnancy-induced hypertension in a group of gestational diabetes women. On the other hand, when compared to placebo, metformin did not exhibit any beneficial effect regarding preeclampsia [48, 49]. In parallel, Pravastatin has been pointed out as a good option to prevent preeclampsia, although larger studies must be done with dose escalation to confirm its effectiveness [50, 51]. Thus, the urgent need to discern, as early as possible, the pregnant woman who is at greater risk of presenting preeclampsia, ideally in its subclinical phase, is outlined, so that it is possible to implement preventive measures. Similar to what has occurred in the last 20 years with the tracing and detection of aneuploidies, in which predictive models were applied in the first trimester in a much more effective way, an efficient and early predictor of preeclampsia is currently being searched within the new inverted pyramid proposal of prenatal care. This proposal suggests that efforts should be concentrated in the first trimester, in order to have an earlier diagnosis and therapeutic proposals established in the subclinical stages of the aggravations [52–54].

## 5. Prediction (Predicting Factors)

Considering the magnitude of the social and economic impact of preeclampsia, in addition to the evident clinical repercussions, there is a need to foresee this condition. Regarding the time when it is possible to predict preeclampsia, although the tendency around the first trimester, some models have been proposed in later gestational ages, based on the fact that a huge proportion of pregnant women need to be reassessed in a late-second or early-third trimester. This proportion of pregnant women may develop preeclampsia after 32 weeks of gestation [13, 55, 56]. However, there is an undeniable concentration of efforts in the first trimester. Over the years, biophysical and biochemical markers were identified as possible early indicators of failures in the complex placentation process, which would lead to preeclampsia. The first step would be to identify the risk factors for the occurrence of this condition.

The risk factors for preeclampsia have been listed as having had preeclampsia in a previous gestation, being nulliparous, being at some age extreme (below 20 years old or above 40), and having African-American descent. Preexisting pathological conditions such as chronic hypertension, diabetes mellitus, nephropathy, and antiphospholipid antibody syndrome are also included in the list [21, 57], as well as BMI above 35 and use of assisted reproductive technologies [58, 59]. The latter is associated with a higher incidence of multiple pregnancies and with an increase in the average age of women in their first pregnancy, which together act to increase the occurrence of preeclampsia [60, 61].

The* National Institute for Health and Clinical Excellence (NICE)* proposed, in a document published in 2010, a classification of the risk factors for preeclampsia as “moderate risk” and “high risk,” so that it would make them tools capable of defining the group for which the immediate application of prophylactic measures would be indicated [62]. The following factors were classified as high risks:

- History of any hypertensive disorder in previous pregnancies;
- Chronic kidney disease;
- Autoimmune diseases such as systemic lupus erythematosus or antiphospholipid antibody syndrome;
- Diabetes type 1 or 2;
- Chronic arterial hypertension.

The following factors were considered as moderate-risk factors:

- Primiparity;
- Women aged 40 years or older;
- Interdelivery interval greater than 10 years;
- Body mass index (BMI) greater than 35 kg/m^2^ at the beginning of prenatal care;
- A family history of preeclampsia;
- Multiple gestations.

According to this institute (NICE), the presence of two moderate-risk factors or a single high-risk factor would admit pregnant women to prophylactic measures (aspirin use before the 16th week of gestation and prenatal care in a specialized service) [46].

For the* American College of Obstetricians and Gynecologists *(ACOG), the risk factors are the same as those reported by the NICE institute [63], with the exception of the BMI, here considered 30 kg/m^2^, the value above which a woman would be at a high risk for preeclampsia. In addition, this institution does not recognize different scales for risk factors; thus they categorize them all under the same denomination of “high risk” [64].

A prospective study applying the concept proposed by the NICE institute to use risk factors as test predictors for preeclampsia obtained a detection rate of 37% and 28.9% of cases in early and late preeclampsia, respectively. It had a rate of 5% of false-positive and it studied a heterogeneous population, composed of nulliparous and multiparous [12]. In this same study, it was demonstrated that the clinical factor of greater predictive capacity was the previous history of preeclampsia. Evidently, this scenario does not favor the identification of nulliparous women at risk for preeclampsia, which constitutes a major limitation, since the incidence of this complication is higher in this group of pregnant women. Specifically for nulliparous women, a multicenter study conducted among more than 8,000 women with low-risk pregnancy demonstrated a 37% detection rate for preeclampsia using a predictor model composed exclusively of clinical factors [14]; obesity and primiparity appeared as the main demographic predictor elements of early preeclampsia.

Blood pressure monitoring is part of a prenatal routine and may be the first clinical occurrence indicator of any hypertensive condition. Considering that the average blood pressure levels are high in pregnant women who will develop preeclampsia in the first or second trimester, or even who already had it elevated before pregnancy [65, 66], this marker ceases to represent a tool for the diagnosis of hypertensive conditions and starts to act as an important predictor of preeclampsia. In a systematic review published in 2008, a higher accuracy of the mean arterial pressure (MAP) in the prediction of preeclampsia among low-risk pregnant women in the second trimester when compared to the isolated measurement of systolic and diastolic blood pressure has been demonstrated. Among women at high risk for preeclampsia, it is the diastolic blood pressure above 75 mmHg that exhibits the greatest predictive capacity of the disease in question [67]. It is necessary to emphasize that the device used in the measurements must be specialized and validated to obtain blood pressure values, and it must be managed by a trained professional. Evaluated in a heterogeneous population composed by nulliparous and multiparous, the prediction rate of the MAP in early and late preeclampsia cases is 58% and 44%, respectively, with 5% of false positives [53].

The Doppler velocimetry study of the uterine arteries provides a noninvasive evaluation of the uteroplacental circulation [53, 68]. As indicated by studies, this resource would be very useful to predict cases of early preeclampsia and, when applied in pregnant women who are considered to be at high risk, to evaluate the development of the disease [69]. When an increase in blood flow resistance in the uterine arteries at 23 weeks of gestation is detected, in a random and heterogeneous population of pregnant women, the sensitivity obtained was of 77.8% and the specificity was of 95% for early preeclampsia prediction [70]. On the other hand, the numbers are not encouraging when the cases of preeclampsia, in general, are evaluated (including those of late manifestation, which are the majority). In these cases, the sensitivity found was of 42.8%, which has no clinical applicability in the case of a screening test [70]. When the Doppler study was early performed in the uterine arteries, between 11 and 13 weeks, and analyzed in an isolated way, the detection rate of early and late preeclampsia was 59% and 40%, respectively, with 5% of false-positive. It is necessary to emphasize that Doppler performance is entirely dependent on the availability of the ultrasound device (which is not always a reality depending on the considered clinical center), on the examiner, and on his or her expertise and training, which restricts its use on a large scale [53]. Considering these variables and the scarcity of existing scientific evidence that showed the improvement in perinatal and maternal outcomes, the Doppler uterine artery study is not recommended as a screening test for preeclampsia among women considered at low risk due to their personal and clinical history [71, 72].

Innumerous biomarkers have been studied, as indicated in a recent systematic review that evaluates inflammatory evidence and their capacity to predict preeclampsia. However, it is still not possible to elect one isolated factor that is sufficient for a prediction of this condition [73]. Table 1 lists some of the most important biomarkers and their accuracy in predicting preeclampsia occurrence, expressed in the area under the ROC curve (AUC). These biomarkers were evaluated before the 16th week of gestation.

It is noteworthy that, when analyzed specifically for cases of early manifestation (before 34 weeks), PAPP-A and PlGF demonstrated better results [74, 75], which draws our attention to the challenge that the preeclampsia prediction may represent, considering its phenotypes variety. Furthermore, the definition of normality parameters for these markers is influenced by the presence or absence of diabetes mellitus, parity (multiparous patients have lower PAPP-A values than nulliparous patients), twinhood (who have higher levels of PAPP-A and PIGF than those observed in single pregnancies), advanced maternal age (women over 35 years of age presented lower values of the markers in question), among other elements [76].

Considering the complexity of the preeclampsia etiology, it is unlikely that an isolated maternal factor will be able to predict this disease. Thus, the tendency worldwide is building algorithms, combining multiple factors. The results are sometimes robust. For instance, a model combining uterine artery Doppler velocimetry, mean arterial blood pressure, and PlGF reached a detection rate of 90% for early-onset cases of preeclampsia [77]. However, when the outcome analyzed is the late-onset cases, which is the vast majority of cases, these numbers stay modest.

For this reason, the need to search for other prediction ways that include different preeclampsia syndrome phenotypes, considering the fact that the cases of late manifestation are the most frequent [78], remains. In this scenario, omics technologies have been pointed out as promising for the identification of early preeclampsia predictors, including a subclinical stage of the disease. Metabolomics is one of these technologies used for the metabolites identification, small molecules that represent the final line of gene expression and a phenotypic signature in high resolution of the disease desired to be studied [79, 80]. There have been efforts to seek a metabolic profile that may be associated with preeclampsia [78, 81–91], but no predictive models have been suggested so far that may have real clinical applicability or that have been validated in large populations. The elucidation of the metabolic profile of preeclampsia will be able to act not only for prediction but also to provide a better understanding of the aggravation with regard to cellular and molecular mechanisms. Ultimately, it may contribute to a better understanding of the disease in all its nuances: prevention, diagnosis, and therapeutic conduction.

## 6. Conclusion

As has been said, preeclampsia is still one of the main causes of death and severe maternal morbidity. The complexity of its pathophysiology is a challenge for future studies and it may help with prevention measures and with the conduction of cases already defined as preeclampsia. Identifying risk groups for preeclampsia through accessible and effective technology, especially in developing countries, can result in better maternal and perinatal public health outcomes since prenatal care would be implemented prior to the establishment of the grievance. To that end, omics technologies have been further studied so that they can broaden the preeclampsia understanding as a whole and, particularly, its prediction.

## Figures and Tables

**Table 1:** Preeclampsia prediction potential of biomarkers and maternal characteristics.

**Biomarker**	**AUC (95**%**CI)**	**Reference**
**Maternal characteristics** **∗**	0.78 (0.71-085)	Goetzinger et al. [12]

**PAPP-A**	0.64 (0.57-0.72)	Goetzinger et al.

**ADAM-12**	0,58 (0.50-0.67)	Goetzinger et al.

**Maternal characteristics ** **+PAPP-A+ADAM-12**	0.79 (0.71-0.86)	Goetzinger et al.

**PlGF**	0.61 (0.56-0.66)	Myatt et al. [13, 14]

**PAPP-A**	0.54 (0.49-0.59)	Myatt et al.

**ADAM-12**	0.58 (0.53-0.63)	Myatt et al.

**Maternal** **characteristics∗∗+PlGF+PAPP-A+ADAM-12**	0.73 (0.69-0.77)	Myatt et al.

**sFlt-1**	0.54 (0.48-0.59)	Myatt et al.

**PP13**	0.51 (0.46-0.56)	Myatt et al.

*∗*Considered maternal characteristics: African-American ethnicity, body mass index, pregestational diabetes mellitus; *∗∗*Considered maternal characteristics: African-American ethnicity, body mass index, systolic blood pressure, and educational level; PAPP-A, pregnancy-associated plasma protein A; ADAM-12, A Disintegrin and Metalloprotease 12; PlGF, placental growth factor; sFlt-1, soluble fms-like tyrosine kinase 1; PP13, placental protein 13; AUC, area under the curve.
